# Finite-Time Controller for Coordinated Navigation of Unmanned Underwater Vehicles in a Collaborative Manipulation Task

**DOI:** 10.3390/s23010239

**Published:** 2022-12-26

**Authors:** Josué González-García, Néstor Alejandro Narcizo-Nuci, Alfonso Gómez-Espinosa, Luis Govinda García-Valdovinos, Tomás Salgado-Jiménez

**Affiliations:** 1 Tecnologico de Monterrey, Escuela de Ingenieria y Ciencias, Av. Epigmenio González 500, Fracc. San Pablo, Queretaro 76130, Mexico; 2 Center for Engineering and Industrial Development-CIDESI, Energy Division, Queretaro 76125, Mexico

**Keywords:** unmanned underwater vehicle, autonomous underwater vehicle, trajectory tracking, finite-time convergence, sliding mode control, collaborative mission, multi-robot

## Abstract

Unmanned underwater vehicles perform inspection and maintenance tasks in complex and changing environments. Some of these tasks require synchronous navigation of multiple vehicles, which is challenging. This paper proposes a synchronous navigation scheme for two BlueROV2 underwater vehicles for a coordinated multi-vehicle task. In the proposed scheme, the vehicles perform the collaborative task of grasping, transporting, and releasing an object. In this scheme, no vehicle-to-vehicle communication is required. A model-free second-order sliding mode controller with finite-time convergence is used to accomplish this task. The controller’s convergence time is user-defined and does not depend on the physical or hydrodynamic parameters of the vehicle, unlike the other finite-time controllers found in the literature. Simulation experiments were conducted to verify the controller’s performance, including high ocean currents as external disturbances. Comparisons were made with two state-of-the-art controllers with finite-time convergence. The results showed that the proposed controller achieved the best results, as the user-defined convergence time was achieved for both vehicles and the collaborative task was completed, no ripples, deviations, or oscillations were observed, and no chattering occurred. The results proved the robustness of the controller in the presence of high ocean currents without the need to readjust the parameters.

## 1. Introduction

### 1.1. Background

Unmanned underwater vehicles (UUVs) have gained importance in science and industry because they can perform some tasks of high risk for humans while reducing mission time and lowering costs [1,2]. They can also perform some other tasks that humans cannot due to the challenging conditions of the underwater environment. In the literature, there are several works in which UUVs are used for data gathering or inspection. However, in recent years, there has been an increased interest in developing UUVs that can perform tasks requiring interaction with underwater objects or the environment [3,4]. 

Some manipulation tasks are so complicated that they require the participation of multiple UUVs in a collaborative scheme [5]. Most of these tasks are for Remotely Operated Vehicles (ROVs), but some work seeks some level of automation. Whether the task is designed for a single vehicle or multiple vehicles, achieving some tasks, such as autonomous navigation, is highly complicated. This is due to nonlinearities in the hydrodynamics of the UUV, uncertainties in the underwater environment, and external disturbances caused by ocean currents and other factors [5]. 

In autonomous navigation, trajectory tracking is the most complex problem because the UUV is expected to follow a temporally and geometrically parameterized trajectory [6]. Several control strategies have been proposed to solve this problem for a single vehicle. The PID controller is the most used due to its simple structure, but it is only effective for specific conditions and not variable conditions. However, when combined with other strategies, better performance can be obtained [7,8,9]. Backstepping [10,11,12] and Neural Networks (NNs) [13,14,15] are other control strategies used for this purpose. Both strategies allow better management of uncertainties and nonlinearities, but they also have weaknesses. Sliding mode control (SMC) has shown excellent performance in the past but has the problem of the chattering effect [16]. 

### 1.2. Related Work

Yu et al. [17] presented a PID controller as a sliding surface of an SMC that results in all tracking errors being confined to a small neighborhood of the origin, even with parameter perturbation and uncertainties in the model. Qiao et al. [18] proposed a second-order adaptive fast non-singular terminal SMC, which resulted in local exponential convergence of position and attitude tracking errors of zero. Chattering was eliminated without reducing the tracking accuracy. Li et al. [19] proposed a backstepping SMC. The controller showed a faster response and shorter adjustment time but with a more significant overshoot. García-Valdovinos et al. [20,21] presented a Model-Free High-Order SMC that reduces the chattering effect by introducing an integral action. They added a backpropagation NN to the model to compensate for perturbations and deal with parametric uncertainties. 

Finite-time controllers have been extensively designed considering other systems [22,23,24,25,26] rather than UUVs. However, a few works presenting an SMC with some form of finite-time convergence for UUVs can be found in the literature. Guerrero et al. [27] presented a high-order adaptive SMC. The real-time experiments showed that the control signal is smoother compared with other controllers. The finite-time convergence of the algorithm is observed, counteracting disturbances. Qiao et al. [28] implemented a double-loop integral terminal SMC with a kinematic and a dynamic loop. The controller tracks the desired trajectory in finite-time even in the presence of uncertainties and external disturbances. Liu et al. [29] proposed a method based on a non-singular terminal SMC manifold. The results showed better performance in convergence rate and tracking accuracy without chattering. González-García et al. [30] implemented a Model-Free High-Order SMC with a time-parameterized gain in the sliding manifold to achieve convergence in finite-time. Simulation results showed up to a 50% reduction in power consumption. The authors validated the proposed controller in experiments with the BuleROV2 vehicle in a swimming pool for the trajectory tracking and station-keeping problem [26,27] and obtained excellent results. Convergence of tracking errors to a practical zero value was achieved in the user-defined time-base for all reported experiments. 

Fewer works can be found in the literature concerning cooperative UUV manipulation tasks. Casalino et al. [31] and Manerikar et al. [32] presented a control algorithm for the cooperative transport of large objects by UUVs. This work was part of the project MARIS, which aimed to develop cooperative control strategies for multiple UUVs. They presented results from numerical simulations that assumed that the object had already been grasped and that information exchange between vehicles was minimal. Simetti et al. [33] developed a unified architecture for controlling single and cooperative UUVs. In support of their proposal, the authors presented a complete simulation of a manipulation task requiring minimal communication. In the simulations, the vehicles approached the object position, then perform the grasp and use the proposed algorithm to move the object to its destination in a defined final position. 

As can be seen, the work found in the literature is usually validated through numerical simulations since it is challenging to assemble an experimental setup with multiple vehicles for this kind of application. Moreover, a simulated environment permits authors to make assumptions about the hardware or software that a real system may not fulfill. A formal hardware or software integration testing method would be necessary to ensure a successful and safe system implementation. A Model-Based Testing (MBT) method [34,35] helps authors identify and assess the threats to the system’s performance, find vulnerabilities, and ensures that the algorithms validated in numerical simulations produce similar results when implemented in real UUVs in real environments. 

Additionally, significant issues, such as vehicle localization, must be solved. Global positioning systems are not available in underwater environments, so the vehicles’ localization must be addressed with acoustic or inertial methods, which are usually slow and not as accurate as needed for collaborative automated manipulation missions. 

Communication is another big issue when working with multiple vehicles in an underwater environment. Radio and other signals commonly used in ground or aerial systems cannot be used underwater. Acoustic communication is used instead. However, it has shortcomings and challenges since it suffers from small bandwidth, propagation delays, and unreliability [36]. Vehicle-to-vehicle (V2V) and vehicle-to-infrastructure (V2I) communications are usually limited to a minimal data exchange to deal with the limitations of the acoustic systems. 

A decentralized scheme based solely on vehicle sensing where no V2V or V2I communications are needed to permit the vehicles to make decisions autonomously [37], enhancing their performance in a task such as the collaborative manipulation of objects in an underwater environment. In this scenario, an autonomous navigation controller with finite-time convergence would have some advantages, such as faster convergence time, higher accuracy, and better anti-disturbance capabilities. If the user could select the convergence time arbitrarily, it would have some extra advantages, such as lower energy consumption and guaranteeing that the vehicle meets and tracks the desired trajectory at a convenient moment.

### 1.3. Contributions

A collaborative task between two UUV is presented in this paper. The proposed scheme for the mission does not consider V2V communication. Instead, it uses a model-free second-order sliding mode control with finite-time convergence to coordinate the two vehicles in the task. The proposed controller converges to a practical zero error in a user-defined time, which guarantees that the vehicles follow a time-parameterized trajectory. The designed task is to approach an object, grab the object, move it to a desired final position, and return to a defined home position. Numerical simulations were performed to evaluate the proposed controller and the collaborative task. The performance of the proposed controller was also compared with two state-of-the-art finite-time controllers. 

The rest of the paper is organized as follows: Section 2 introduces UUV modeling and describes the proposed controller and the BlueROV2 vehicle considered for this work. Section 3 presents the design of the collaborative manipulation task and the methodology for its validation. Section 4 presents the results of the numerical simulations, and Section 5 summarizes the general conclusions and describes future work.

## 2. Materials and Methods

This section introduces the modeling of UUV, its kinematics, and hydrodynamics. Then, the proposed model-free high-order SMC is presented, describing its design, stability analysis, and finite-time convergence feature. Finally, the BlueROV2 vehicle is described and its model is defined, which is used for the Matlab/Simulink numerical simulator.

### 2.1. Unmanned Underwater Vehicle Model

**Figure 1:**
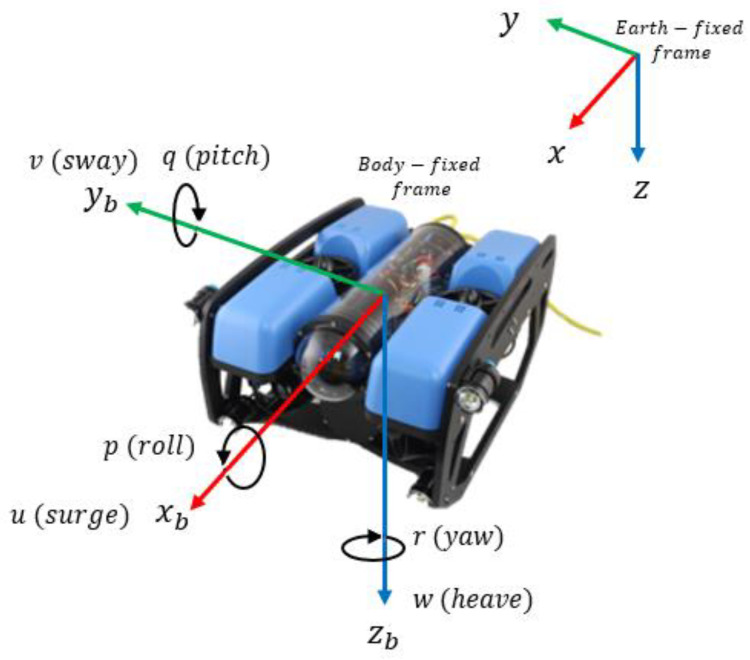
Reference frames for an unmanned underwater vehicle.

Two reference frames are needed to describe a UUV [38]: an Earth-fixed frame, known as North-East-Down (NED), and a Body-fixed frame, located at the center of buoyancy of the vehicle, as shown in Figure 1.

The Society of Naval Architects and Marine Engineers (SNAME) [39] has established a notation referring to the positions, velocities, forces, and moments acting on a UUV. This notation is shown in Table 1.

Then, a position vector can be expressed in the Earth-fixed frame as

(1) $$\eta ={\left(\eta_{1},\eta_{2}\right)}^{T}={\left(x,y,z,\phi ,\theta ,\psi \right)}^{T},\;$$

and a general velocity vector can be expressed in the Body-fixed frame as

(2) $$\nu ={\left(\nu_{1},\nu_{2}\right)}^{T}={\left(u,v,w,p,q,r\right)}^{T}.$$

#### 2.1.1. Kinematic Model

A transformation from the Body-fixed frame to the Earth-fixed frame can be performed by applying the following expression

(3) $$\dot{\eta }=J\left(\eta_{2}\right)\nu ,$$

where $J\left(\eta_{2}\right)$ is a transformation matrix defined as

(4) $$J\left(\eta_{2}\right)=\left[\begin{array}{cc} J_{1}\left(\eta_{2}\right) & 0_{3\times 3}\\ 0_{3\times 3} & J_{2}\left(\eta_{2}\right) \end{array}\right],$$

where $\eta_{2}=\left[\phi ,\theta ,\psi \right]$, $J_{1}\;$is the rotation matrix that provides the component of the linear velocities in the Earth-fixed frame, and $J_{2}$ is the matrix that relates angular velocities with the vehicle’s velocities in the Earth-fixed frame. These matrices are defined as

(5) $$J_{1}\left(\eta_{2}\right)=\left[\begin{array}{ccc} c_{\theta }c_{\psi } & -s_{\psi }c_{\phi }+s_{\phi }s_{\theta }c_{\psi } & s_{\phi }s_{\psi }+s_{\theta }c_{\phi }c_{\psi }\\ s_{\psi }c_{\phi } & c_{\phi }c_{\psi }+s_{\phi }s_{\theta }s_{\psi } & -c_{\phi }c_{\psi }+s_{\theta }s_{\psi }c_{\phi }\\ -s_{\theta } & s_{\phi }c_{\theta } & c_{\phi }c_{\theta } \end{array}\right],$$

(6) $$J_{2}\left(\eta_{2}\right)=\left[\begin{array}{ccc} 1 & s_{\phi }t_{\theta } & c_{\phi }t_{\theta }\\ 0 & c_{\phi } & -s_{\phi }\\ 0 & \frac{s_{\phi }}{c_{\theta }} & \frac{c_{\phi }}{c_{\theta }} \end{array}\right],$$

where $c=\cos\left(a\right),\;s=\sin\left(a\right),$ and $t=\tan\left(a\right)$.

**Assumption** **1.** *The pitch angle θ is bounded such that $\left|\theta \right|<\theta_{M}<\pi /2$ to avoid any possible singularity problem of $J\left(\eta_{2}\right)$. Here, $\theta_{M}$ stands as the upper bound of θ and it is a known positive constant.*

**Remark** **1.** *Well-posed Jacobian matrix. The transformation in Equation (3) is ill-posed when θ=±90°. A quaternion approach might be considered to overcome this singularity. However, the vehicle is not expected to operate at θ=±90°. In addition, the UUV is utterly stable in roll and pitch coordinates.*

Based on Assumption 1, the following assumption can be made

**Assumption** **2.** *The Jacobian transformation matrix $J\left(\eta_{2}\right)$ is bounded by a known positive constant $J_{sup}$* [28] *such that $sup_{\eta }∥J\left(\eta \right)∥\leq J_{sup}$.*

#### 2.1.2. Hydrodynamical Model

The hydrodynamical model for a UUV was proposed by Fossen [38] considering 6 Degrees of Freedom (DoF) as

(7) $$M\dot{\nu }+C\left(\nu \right)\nu +D\left(\nu \right)\nu +g\left(\eta \right)=\tau_{\upsilon },$$

where M ∈ℝ^6×6^ is the inertial and added mass matrix, C ∈ℝ^6×6^ is the rigid body and added mass centripetal and Coriolis matrix, D ∈ℝ^6×6^ is the hydrodynamic damping matrix, g ∈ℝ^6×1^ is the restitution forces vector, and $\tau_{\upsilon }$ ∈ℝ^6×1^ represents the control input vector.

The model presented in Equation (7) can be expressed in the Earth-fixed frame by applying the kinematic transformations

(8) $$\dot{\eta }=J\left(\eta_{2}\right)\nu ↔\;\nu =J^{-1}\left(\eta_{2}\right)\dot{\eta },$$

(9) $$\ddot{\eta }=J\left(\eta_{2}\right)\dot{\nu }+\dot{J}\left(\eta_{2}\right)\nu ↔J^{-1}\left(\eta_{2}\right)\left[\ddot{\eta }-J\left(\eta_{2}\right)\nu \right].$$

After some manipulation, the model is transformed into the Earth-fixed frame as

(10) $$M_{\eta }\left(\eta \right)\ddot{\eta }+C_{\eta }\left(\nu ,\eta \right)\dot{\eta }+D_{\eta }\left(\nu ,\eta \right)\dot{\eta }+g_{\eta }\left(\eta \right)=\tau_{\eta }$$

with

(11a) $$M_{\eta }\left(\eta \right)=J^{-T}\left(\eta \right)MJ^{-1}\left(\eta \right),$$

(11b) $$C_{\eta }\left(\nu ,\eta \right)=J^{-T}\left(\eta \right)\left[C\left(\nu \right)-MJ^{-1}\left(\eta \right)\dot{J}\left(\eta \right)\right]J^{-1}\left(\eta \right),$$

(11c) $$D_{\eta }\left(\nu ,\eta \right)=J^{-T}\left(\eta \right)D\left(\nu \right)\;J^{-1}\left(\eta \right),$$

(11d) $$g_{\eta }\left(\eta \right)=J^{-T}\left(\eta \right)g\left(\eta \right),$$

(11e) $$\tau_{\eta }=J^{-T}\left(\eta \right)\tau_{\upsilon }.$$

The model provided in Equation (10) holds the following properties

**Property** **1.** *The inertia Matrix$\;M_{\eta }\left(\eta \right)$ is symmetric and positive definite, i.e., $M_{\eta }\left(\eta \right)=M_{\eta }{\left(\eta \right)}^{T}>0$, ${\dot{M}}_{\eta }\left(\eta \right)=0$.*

**Property** **2.** *The derivative of the inertia matrix ${\dot{M}}_{\eta }\left(\eta \right)$ and the Coriolis and centripetal matrix $C_{\eta }\left(\nu ,\eta \right)$ satisfy $x^{T}\left[{\dot{M}}_{\eta }\left(\eta \right)-2C_{\eta }\left(\nu ,\eta \right)\right]x=0,\;\forall x\in {\mathbb{R}}^{n},\;x\neq 0$. Thus ${\dot{M}}_{\eta }\left(\eta \right)-2C_{\eta }\left(\nu ,\eta \right)$ is skew-symmetric.*

**Property** **3.** *The damping matrix $D_{\eta }\left(\nu ,\eta \right)$ is non-symmetric and strictly positive, i.e., $D_{\eta }\left(\nu ,\eta \right)>0,\;\forall \;\upsilon ,\eta \in {\mathbb{R}}^{n}$.*

**Property** **4.** *The dynamic model of a UUV is linearly parametrizable by the product of a regressor $Y\left(\eta ,\dot{\eta },\ddot{\eta }\right)\in {\mathbb{R}}^{n\times p}$ composed of known functions, and a vector $\theta \in {\mathbb{R}}^{p}\;$composed of dynamic parameters. That is $Y\left(\eta ,\dot{\eta },\ddot{\eta }\right)\theta \in {\mathbb{R}}^{n}$.*

**Property** **5.** *Boundedness of dynamic terms. For constant$\;\beta_{i}>0$*

*a.*  *The inertia matrix $M_{\eta }\left(\eta \right)$ satisfies $\beta_{1}<\lambda_{m}\left(M_{\eta }\left(\eta \right)\right)\leq ∥M_{\eta }\left(\eta \right)∥\leq \lambda_{M}\left(M_{\eta }\left(\eta \right)\right)<\beta_{2}$ with $\lambda_{m}$ and $\lambda_{M}\;$denoting the minimum and maximum eigenvalues of M, respectively;*
*b.*  *The Coriolis and centripetal vector $C_{\eta }\left(\nu ,\eta \right)\dot{\eta }$ satisfies $∥C_{\eta }\left(\nu ,\eta \right)\dot{\eta }∥\leq \beta_{3}∥\nu ∥∥\eta ∥,\forall \;\nu ,\eta \in {\mathbb{R}}^{n}$;*
*c.*  *The damping vector $D_{\eta }\left(\nu ,\eta \right)\dot{\eta }$ satisfies $∥D_{\eta }\left(\nu ,\eta \right)\dot{\eta }∥\leq \beta_{4}∥\nu ∥∥\eta ∥,\forall \;\nu ,\eta \in {\mathbb{R}}^{n}$;*
*d.*  *The vector of restoring forces $g\left(\eta \right)$ satisfies the following $∥g\left(\eta \right)∥<\beta_{5}$.*

### 2.2. Model-Free High Order Sliding Mode Control

García-Valdovinos et al. [21] established that the left side of Equation (10) is linearly parameterizable by the product of a regressor $Y\left(\eta ,\dot{\eta },\ddot{\eta }\right)$ composed of known functions, and a vector $\theta \in {\mathbb{R}}^{p}$ composed of parameters (Property 4). The parameterization $Y\left(\eta ,\dot{\eta },\ddot{\eta }\right)\theta$ can be written in terms of a nominal reference ${\dot{\eta }}_{r}$, and its time derivative ${\ddot{\eta }}_{r}$ is as follows

(12) $$M_{\eta }\left(\eta \right){\ddot{\eta }}_{r}+C_{\eta }\left(\nu ,\eta \right){\dot{\eta }}_{r}+D_{\eta }\left(\nu ,\eta \right){\dot{\eta }}_{r}+g_{\eta }\left(\eta \right)=Y\left(\eta ,\dot{\eta },{\dot{\eta }}_{r},{\ddot{\eta }}_{r}\right)\theta .\;$$

Subtracting Equation (12) from both sides of Equation (10) results in

(13) $$M_{\eta }\left(\eta \right){\dot{S}}_{r}+C_{\eta }\left(\nu ,\eta \right)S_{r}+D_{\eta }\left(\nu ,\eta \right)S_{r}=\tau_{\eta }-Y\left(\eta ,\dot{\eta },{\dot{\eta }}_{r},{\ddot{\eta }}_{r}\right)\theta ,\;$$

where $S_{r}=\dot{\eta }+{\dot{\eta }}_{r}$ is defined as the extended error, ${\dot{S}}_{r}=\ddot{\eta }+{\ddot{\eta }}_{r}$ denotes its time derivative, and the nominal reference ${\dot{\eta }}_{r}$ is defined as follows

(14) $${\dot{\eta }}_{r}={\dot{\eta }}_{d}-\alpha \tilde{\eta }+S_{d}-K_{i}\int_{0}^{t}sign\left(S_{\eta }\right)d\sigma ,$$

where $\tilde{\eta }=\eta -\eta_{d}\;$represents the position tracking error, $\eta_{d}$ denotes the desired positions, $K_{i}\;$is a diagonal positive definite n×n gain matrix, and α>0 is a gain to be defined, $sign\left(x\right)$ stands for the entry-wise sign function of vector x. Now, consider

(15) $$S=\dot{\tilde{\eta }}+\alpha \tilde{\eta },$$

(16) $$S_{d}=S\left(t_{0}\right)e^{-kt},$$

(17) $$S_{\eta }=S-S_{d},$$

with κ>0.

**Remark** **2.** *Since $e^{-kt}$ serves as an input of $\dot{\tilde{\eta }}+\alpha \tilde{\eta }$, a vanishing function $S_{d}$ is used to make $S_{\eta }=S\left(0\right)-S_{d}=0$ for t=0. The term $S_{d}$ aims to improve the transient response and does not affect the stability or the boundedness of the controller’s signals. After a brief period slightly greater than zero, $S_{\eta }$ becomes S and would be bounded for all time.*

The extended error $S_{r}=\dot{\eta }-{\dot{\eta }}_{r}$ can be written as

(18) $$S_{r}=S_{\eta }+K_{i}\;\int_{0}^{t}sign\left(S_{\eta }\right)d\sigma \;,$$

and its time derivative ${\dot{S}}_{r}$ as

(19) $${\dot{S}}_{r}={\dot{S}}_{\eta }+K_{i}sign\left(S_{\eta }\right).\;$$

**Assumption** **3.** *The nominal reference ${\dot{\eta }}_{r}$ and its time derivative ${\ddot{\eta }}_{r}$ are bounded by positive scalars $\beta_{i},\;i=6,\ldots ,9$ as follows*

(20) $$\|{\dot{\eta }}_{r}\|\leq \beta_{6}+\|\alpha \|\;\|\tilde{\eta }\|+\lambda_{M}\left(K_{i}\right)I<\beta_{7},$$

(21) $$\|{\ddot{\eta }}_{r}\|\leq \beta_{6}+\|\alpha \|\;\|\dot{\tilde{\eta }}\|<\beta_{9}.\;$$

Finally, the control law is provided by

(22) $$\tau_{\eta }=-K_{d}S_{r},$$

where $K_{d}$ is a diagonal definite positive n×n gain matrix.

**Remark** **3.** *Note that the controller does not require knowledge of the vehicle’s parameters or hydrodynamics.*

Finally, to apply the control signals in the vehicle, the following transformation must be applied

(23) $$\tau_{\upsilon }=J^{-1}\left(\eta_{2}\right)\tau_{\eta }.\;$$

#### 2.2.1. The Feedback Gain *α*(*t*)

The control law presented in Equation (22) conduces to a second-order SMC with asymptotic convergence when the value of the α gain from Equation (15) is set to a constant [21]. By replacing this constant gain with a time-parametrized $\alpha \left(t\right)$ gain it is possible to achieve a finite-time convergence. To this end, $\alpha \left(t\right)$ is defined using a Time-Base Generator (TBG)

(24) $$\xi \left(t\right)=10\;\frac{{\left(t-t_{0}\right)}^{3}}{{\left(t_{b}-t_{0}\right)}^{3}}-15\frac{{\left(t-t_{0}\right)}^{4}}{{\left(t_{b}-t_{0}\right)}^{4}}+6\frac{{\left(t-t_{0}\right)}^{5}}{{\left(t_{b}-t_{0}\right)}^{5}}\;,$$

which provides a smooth 0 to 1 transition. A time-base parameter defines the convergence time for the controller $t_{b}$, which can be arbitrarily set by the user, resulting in $\xi \left(t_{0}\;\right)=0,\;\xi \left(t_{b}\right)=1$. The time derivative of the TBG is defined as

(25) $$\dot{\xi }\left(t\right)=30\;\frac{{\left(t-t_{0}\right)}^{2}}{{\left(t_{b}-t_{0}\right)}^{3}}-60\frac{{\left(t-t_{0}\right)}^{3}}{{\left(t_{b}-t_{0}\right)}^{4}}+30\frac{{\left(t-t_{0}\right)}^{4}}{{\left(t_{b}-t_{0}\right)}^{5}},$$

it has a bell-shaped profile which results in $\dot{\xi }\left(t_{0}\right)=\dot{\xi }\left(t_{b}\right)=0$.

Then, the time-varying gain $\alpha \left(t\right)$ can be computed as

(26) $$\alpha \left(t\right)=\left\{\begin{array}{cc} \alpha_{0}\frac{\xi \left(t\right)}{\left(1-\xi \left(t\right)\right)+\delta }, & 0\leq t\leq t_{b}\\ \alpha_{c}, & t>t_{b}, \end{array}\right.,$$

where $\alpha_{0}\;\approx \;1,\;0<\delta \;“1,$ and $\alpha_{c}>0$ are constants.

Then, the solution of the differential equation provided in Equation (15) is

(27) $$\tilde{\eta }\left(t\right)=\tilde{\eta }\left(t_{0}\right){\left[1-\xi \left(t\right)+\delta \right]}^{\alpha_{0}}.\;$$

When $t=t_{b}$, then $\xi \left(t_{b}\right)=1$ the solution becomes

(28) $$\tilde{\eta }\left(t_{b}\right)=\tilde{\eta }\left(t_{0}\right)\delta^{\alpha_{0}}.\;$$

**Remark** **4.** *The time-base parameter $t_{b}$ can be directly and arbitrarily set by the user and does not depend on the system’s initial condition or other controller parameters.*

#### 2.2.2. Stability Analysis

**Theorem** **1.** *Consider the control law in Equation (22) in a closed loop with the system model described in Equation (13), which yields*

(29) $$M_{\eta }\left(\eta \right){\dot{S}}_{r}=-K_{d}S_{r}-C_{\eta }\left(\nu ,\eta \right)S_{r}-D_{\eta }\left(\nu ,\eta \right)S_{r}-Y\left(\eta ,\dot{\eta },{\dot{\eta }}_{r},{\ddot{\eta }}_{r}\right)\theta ,$$

*Finite-time tracking of position errors in a very small neighborhood is guaranteed if $K_{d}$ and $K_{i}$ are large enough for small initial error conditions.*

**Proof** **of** **Theorem** **1.** The stability proof was divided into two parts for better comprehension.

Part I. Boundedness of the closed-loop trajectories: Let us consider the following Lyapunov candidate function

(30) $$V=\frac{1}{2}S_{r}^{T}M_{\eta }\left(\eta \right)S_{r},$$

whose time derivative yields

(31) $$\dot{V}=-S_{r}^{T}K_{d}S_{r}-S_{r}^{T}D_{\eta }\left(\nu ,\eta \right)S_{r}-Y\left(\eta ,\dot{\eta },{\dot{\eta }}_{r},{\ddot{\eta }}_{r}\right)\theta ,\;\leq -S_{r}^{T}K_{d}S_{r}-∥S_{r}∥∥Y\left(\eta ,\dot{\eta },{\dot{\eta }}_{r},{\ddot{\eta }}_{r}\right)\theta ∥\;\leq -S_{r}^{T}K_{d}S_{r}-∥S_{r}∥\rho \left(t\right)\;\leq -\lambda_{m}\left(K_{d}\right)∥S_{r}{∥}^{2}+∥S_{r}∥\rho \left(t\right)$$

where arguments were omitted for simplicity, and the skew-symmetric property provided in Property 2 was applied. Moreover, the norm of $Y\left(\eta ,\dot{\eta },{\dot{\eta }}_{r},{\ddot{\eta }}_{r}\right)\theta$ was substituted by an upper bound defined by a state-dependent function $\rho \left(t\right)$. It can be proven that $Y\left(\eta ,\dot{\eta },{\dot{\eta }}_{r},{\ddot{\eta }}_{r}\right)\theta$ is upper bounded [40] since both desired trajectories and UUV dynamics are bounded (according to Property 5 and Assumption 3, there exist upper bounds for$\;M_{\eta },C_{\eta },D_{\eta },g_{\eta },{\dot{\eta }}_{r},{\ddot{\eta }}_{r}$). If $K_{d}$ is large enough and the initial error is small enough, one concludes the negative definiteness of Equation (31) outside of the small ball $\varepsilon_{0}=\left\{S_{r}\left|\dot{V}\geq 0\right.\right\}$ centered at the origin $\dot{V}\left(S_{r}\right)=0$. This boundedness in the $L_{\infty }$ sense leads to the existence of the constant $\varepsilon_{1}>0\;$such that

(32) $$||{\dot{S}}_{r}||\leq \varepsilon_{1}.\;$$

At this point, the stability of the tracking errors was proven with all the closed-loop signals bounded. 

Part II. Existence of the second-order sliding mode: Let us consider the following second-order dynamical system, defined by the time derivative of the nominal reference provided in Equation (19) as

(33) $${\dot{S}}_{\eta }=-K_{i}sign\left(S_{\eta }\right)+S_{r}.$$

Now, considering the product of Equation (33) and $S_{\eta }^{T}$, applying the bound provided by Equation (32), and considering $\mu =\lambda_{m}\left(K_{i}\right)-\varepsilon_{1}\;$results in

(34) $$S_{\eta }^{T}{\dot{S}}_{\eta }=-K_{i}S_{r}^{T}sign(S_{\eta })+S_{\eta }^{T}{\dot{S}}_{r}\;\leq -\lambda_{m}\left(K_{i}\right)|S_{\eta }^{T}|+|S_{\eta }^{T}|∥{\dot{S}}_{r}∥,\;\leq \left|S_{\eta }^{T}\right|\left(-\lambda_{m}\left(K_{i}\right)+\varepsilon_{1}\right),\;\leq -\mu \left|S_{\eta }^{T}\right|.$$

The sliding mode condition is achieved if $\lambda_{m}\left(K_{i}\right)>\varepsilon_{1}$ and then μ>0 guarantees the sliding mode at $S_{\eta }=0$ at $t_{g}=\frac{\left|S_{\eta }\left(t_{0}\right)\right|}{\mu }$. Recall that for any initial condition$\;S_{\eta }\left(t_{0}\right),$ $t_{g}=0$, implying that the sliding mode is enforced for all time and thus

(35) $$S_{\eta }=\dot{\tilde{\eta }}+\alpha \tilde{\eta }=0↔\;\dot{\tilde{\eta }}=-\alpha \tilde{\eta }.$$

which implies that the tracking errors converge to a very small ball centered at the origin ($\tilde{\eta },\dot{\tilde{\eta }})=0$ in a finite time $t_{b}$, according to the solution provided in Equations (27) and (28).

**Remark** **5.** *Practical zero error: Considering the solution of Equation (35) at $t=t_{b}$, $\tilde{\eta }\left(t_{b}\right)=\tilde{\eta }\left(t_{0}\right)\delta^{\alpha_{0}}$, with $\alpha_{0}$ very close to 1 and δ chosen very small, tracking errors belong to a very small vicinity $\epsilon_{2}$ of the origin, which may represent the required accuracy or practical zero error. Note that at$\;t>t_{b}$, the time-varying feedback gain $\alpha \left(t\right)$ must be reset to a desired constant $\alpha_{c}>0$. Since a sliding mode is enforced at all times, $\tilde{\eta }\left(t\right)\in \varepsilon_{2},\;\forall \;t>t_{b}$ and furthermore$\;\tilde{\eta }\left(t\right)$ converges exponentially since $\dot{\tilde{\eta }}=-\alpha_{c}\tilde{\eta }\left(t\right),\;\forall \;t>t_{b}$; therefore, it implies that the tracking errors tend to zero very fast, that is $\eta \to \eta_{d},\dot{\eta }\to {\dot{\eta }}_{d},\forall \;t>t_{b}$.*

**Remark** **6.** *Tuning of parameters $\alpha_{0}$, δ: It can be observed from Equation (27) that, given an initial error condition $\tilde{\eta }\left(t_{0}\right)=0$, the error $\tilde{\eta }\left(t\right)$ is 0 no matter the value of $\alpha_{0}$ and δ, since the vehicle is already in the desired trajectory. For the cases where $\tilde{\eta }\left(t_{0}\right)\neq 0$, a simple way to tune these parameters is by fixing either $\alpha_{0}$ around 1 (slightly greater or smaller than 1) or δ>0 very close to 0. Then, given the initial error condition $\tilde{\eta }\left(t_{0}\right)$, the desired practical zero error $\tilde{\eta }\left(t\right)$ at $t=t_{b}$, and by fixing δ in Equation (28), $\alpha_{0}$ can be computed as*

(36) $$\alpha_{0}=\log_{\delta }\left(\frac{\tilde{\eta }\left(t_{b}\right)}{\tilde{\eta }\left(t_{0}\right)}\right),$$

*or, by fixing $\alpha_{0}$, δ can be computed as follows*

(37) $$\delta =\sqrt[{\alpha_{0}}]{\left(\frac{\tilde{\eta }\left(t_{b}\right)}{\tilde{\eta }\left(t_{0}\right)}\right)}.$$

*Notice that the practical zero error $\tilde{\eta }\left(t_{b}\right)$ is limited to the accuracy and resolution of the measurement sensors. *□

**Figure 2:**
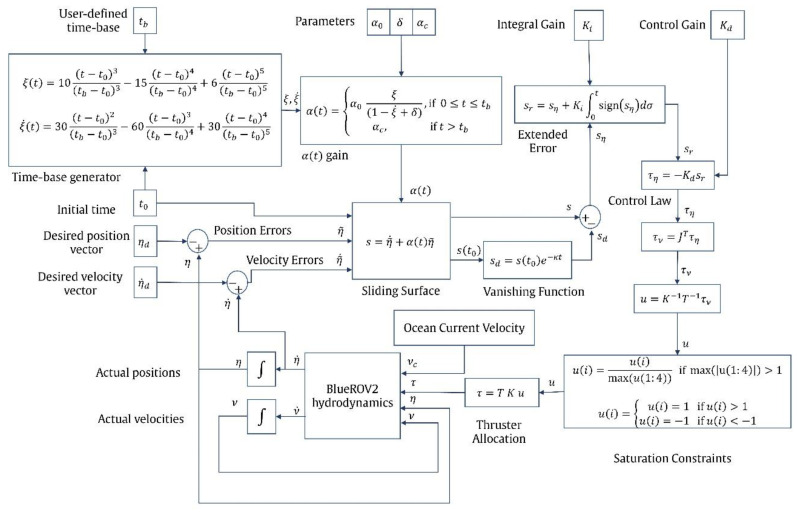
Model-free second-order sliding mode control with finite-time convergence complete block diagram.

A complete block diagram of the proposed controller is shown in Figure 2.

### 2.3. BlueROV2 Vehicle

**Figure 3:**
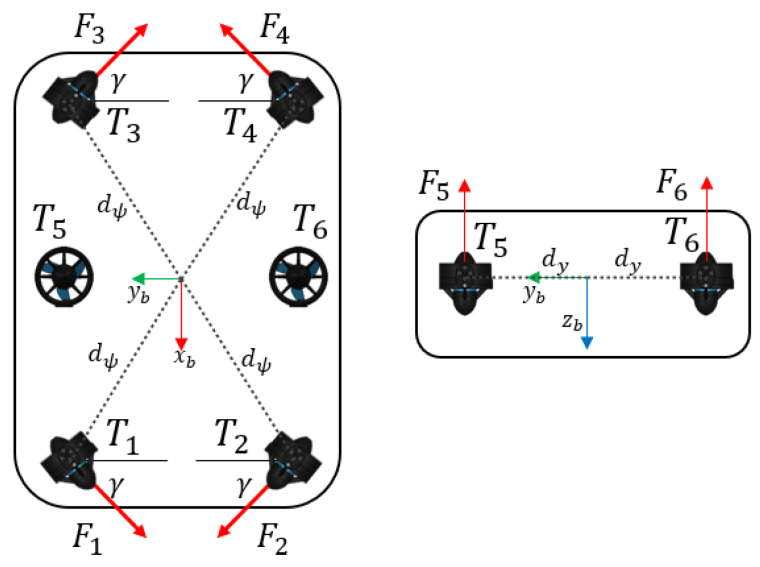
Thruster configuration of the BlueROV2 vehicle. Top view (**left**). Front view (**right**).

The vehicle considered for this work is the BlueROV2 by BlueRobotics^®^. It has six thrusters: two verticals and four horizontals in a vectored arrangement, as shown in Figure 3.

The control signal $\tau_{\upsilon }$ can be computed as

(38) $$\tau_{\upsilon }=T\;K\;u,$$

where u is the thruster control inputs vector, K is a diagonal matrix with the maximum thruster forces (~40 N), and T is the thruster allocation matrix provided by

(39) $$T=\left[\begin{array}{cccccc} 0.7071 & 0.7071 & -0.7071 & -0.7071 & 0 & 0\\ -0.7071 & 0.7071 & -0.7071 & 0.7071 & 0 & 0\\ 0 & 0 & 0 & 0 & -1 & -1\\ 0 & 0 & 0 & 0 & 0.115 & -0.115\\ 0 & 0 & 0 & 0 & 0 & 0\\ -0.1773 & 0.1773 & -0.1773 & 0.1773 & 0 & 0 \end{array}\right]$$

According to [30], the following assumptions apply to the BlueROV2:

1. The vehicle travels at relatively low speeds (i.e., less than two m/s) so that the buoyancy forces can be neglected;
2. The vehicle’s center of gravity (CG) is assumed to be at the intersection of the planes of symmetry;
3. The pitch orientation θ is assumed to be passively stable since the vehicle’s thruster allocation does not allow active control of this DoF.

As mentioned in Remark 3, the proposed controller does not require any hydrodynamic or other vehicle parameters. However, a complete set of BlueROV2 parameters is required for simulation purposes. These parameters are in Table 2, Table 3, Table 4 and Table 5 [41], as used in the Matlab/Simulink numerical simulator used in this work.

## 3. Validation Setup

This section describes the collaborative manipulation task and the validation considerations of the proposed controller for vehicle coordination. Then, two state-of-the-art finite-time controllers for UUV navigation are briefly described.

### 3.1. Design of the Task

A collaborative manipulation task was designed to validate the proposed controller’s performance in such a task. Due to the no communication restriction, the collaboration between vehicles must be achieved by coordinating their navigation to accurately follow a set of predefined trajectories. In this task, two BlueROV2 vehicles approach an object whose position on the bottom of a water tank is known beforehand. Then, the BlueROV2 vehicles grab the object, lift it, and transport it to a predetermined destination to release it. Both vehicles must converge to their trajectories in a shorter time than specified for grabbing the object. The vehicles must maintain a constant distance from each other when carrying the object, and they must face each other. The following are all considerations for the collaborative manipulation task:

- A water tank of 6 m×6 m×3 m is considered the working space. It is considered clear of obstacles or entities other than the two BlueROV2 and the object to manipulate;
- The initial position for the BlueROV2 vehicles is irrelevant for control and does not affect the convergence time;
- An ideal abstract model of the object to be manipulated is considered. Its physical and dynamic properties are neglected;
- The position of the object is known beforehand;
- Grasping the object is considered solved since it is out of the scope of this work. The grasping occurs when the vehicles reach their respective grasping positions;
- The vehicles grasp the object from opposite sides, i.e., they should align their orientations to face each other and maintain a distance of 50 cm;
- There is no communication between the vehicles;

The design of the collaborative task considers nine intervals as follows:

- Interval 1: t≤6 s
- Approach: The vehicles start from an arbitrary initial position and approach the object. The initial position of the object is provided by $Oi\left(1.5,\;3.5,\;3\right)$. The vehicles reach a position 1 m above Oi with a distance of 1 m in the *y*-axis measured from their Body-fixed-frame origin. The *x*-axis coordinate corresponds to the corresponding grasp point. The final orientations of the vehicles are provided by $\psi_{1}=-\frac{\pi \;}{2}\;\mathrm{rad}$ and $\psi_{2}=\frac{\pi }{2\;}\;\mathrm{rad}$;
- Interval 2: 6 s<t≤9 s
- Descending: The vehicles descend to z=3 m;
- Interval 3: 9 s<t≤12 s
- Grasping: The vehicles move slowly along the y-axis and reach their respective grasping points. Grasping of the object takes place;
- Interval 4: 12 s<t≤15 s
- Lifting: The vehicles rise to z=2 m to lift the object from the bottom of the water tank;
- Interval 5: 15 s<t≤21 s
- Transporting: The vehicles coordinately move the object to a position 1 m above the releasing point, defined as $O_{f}=\left(4.5,1.5,3\right)$;
- Interval 6: 21 s<t≤24 s
- Descending: The vehicles lower the object at its final position $O_{f}$;
- Interval 7: 24 s<t≤27 s
- Release: The vehicles release the object and move 0.5 m on the *y*-axis to gain a slight separation from it;
- Interval 8: 27 s<t≤30 s
- Ascending: The vehicles move to z=2 m to gain separation from the object;
- Interval 9: 30 s<t≤40 s;
- Return: The vehicles move to their home positions at opposite corners of the water tank.

**Figure 4:**
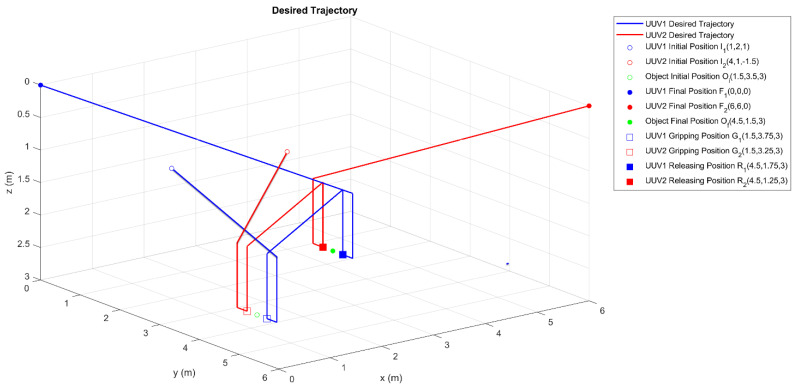
Collaborative manipulation task. Trajectory design for both BlueROV2 vehicles.

All intervals are parameterized using the TBG provided in Equation (24) to ensure smooth and coordinated movement of the vehicles. The velocities are parameterized by Equation (25), which guarantees that the initial and final velocities in each interval are $0\frac{m}{s}$. The complete set of trajectories is shown in Figure 4. An animated MATLAB plot for these trajectories can be found as supplementary material Video S1.

### 3.2. State-of-the-Art Finite-Time Controllers 

For completeness, in addition to the proposed model-free second-order SMC (MFSOSMC), two relevant state-of-the-art controllers [29,42] that address finite-time tracking for UUV are used. All three controllers were evaluated in the simulations for comparative analysis. A brief introduction to these controllers is provided in the following subsections. 

#### 3.2.1. Non-Singular Terminal Sliding Mode Control

Liu et al. [29] presented a non-singular terminal SMC (NSTSMC). This controller includes a non-singular sliding manifold defined by

(40) $$s=e_{2}+\beta F_{f}\left(e_{1}\right),$$

with

(41) $$e_{1}=\eta +\eta_{d},\;$$

(42) $$e_{2}=\dot{\eta }+{\dot{\eta }}_{d},$$

where $\eta_{d}$ and ${\dot{\eta }}_{d}$ are the desired positions and velocities, β is a positive constant and $F_{f}\left(e_{1}\right)$ is provided by

(43) $$F_{f}\left(e_{1}\right)=\left\{\begin{array}{c} \begin{array}{cc} \sqrt{\left|A\left(e_{1}\right)\right|}\frac{{\left(I+e^{-e_{1}}\right)}^{2}}{e^{-e_{1}}}sign\left(e_{1}\right) & if\;\left|e_{1}\right|\geq \epsilon_{1} \end{array}\\ \begin{array}{cc} A_{1}\sin\left(\frac{\pi e_{1}}{2\epsilon_{1}}\right)+\frac{\epsilon_{1}}{2\pi }A_{2}\sin\left(2\pi \frac{e_{1}}{\epsilon_{1}}\right) & if\;\left|e_{1}\right|\leq \epsilon_{1} \end{array} \end{array}\right.,$$

where $\epsilon_{1}$ is a vector of constants, I is a vector of ones with compatible dimensions, $\left|e_{1}\right|$ is the absolute value of each element of $e_{1},$ and

(44) $$A(e_{1})=\frac{I-e^{-e_{1}}}{I+e^{-e_{1}}},\;$$

(45) $$A_{1}=\sqrt{\left|A\left(e_{1}\right)\right|}\frac{{\left(I+e^{-\epsilon_{1}}\right)}^{2}}{e^{-\epsilon_{1}}},$$

(46) $$A_{2}=\frac{1}{\sqrt{\left|A\left(e_{1}\right)\right|}}+\sqrt{\left|A\left(e_{1}\right)\right|}\frac{I-e^{-2\epsilon_{1}}}{e^{-\epsilon_{1}}}.$$

Considering a UUV subject to model uncertainty and external disturbances, the action of control law with the sliding manifold and the adaptive rate is

(47) $$u=u_{eq}+u_{ad},\;$$

(48) $$u_{eq}=B^{+}J^{T}\left(\left({\hat{C}}_{RB\eta }+{\hat{C}}_{\eta }\right)\dot{\eta }+{\hat{D}}_{\eta }\dot{\eta }+{\hat{g}}_{\eta }\right)+B^{+}J^{T}{\hat{M}}_{\eta }\left({\dot{\eta }}_{d}-\beta \frac{\partial F_{f}\left(e_{1}\right)}{\partial e_{1}}\right),$$

(49) $$u_{ad}=-B^{+}J^{T}{\hat{M}}_{\eta }\left(\alpha_{1}s+\alpha_{2}{\left|s\right|}^{\gamma }sign\left(s\right)+\frac{{\hat{L}}_{m}^{T}\Theta }{2\epsilon_{0}}s\right),\;$$

with

(50) $${\dot{\hat{L}}}_{m}=\lambda_{m}^{-1}\left(\frac{∥s^{2}∥}{2\epsilon_{0}^{2}}\Theta -k_{m}^{2}{\hat{L}}_{m}\right),$$

(51) $${\dot{k}}_{m}=-\lambda_{p}^{-1}k_{m},\;$$

where the superscript + denotes a pseudo-inverse operation; α1 and α2 are diagonal matrices; ϵ0 is a positive constant; and γ is a constant between 0 and 1 and

(52) $$\Theta ={\left[1;∥\dot{\eta }∥;∥\dot{\eta }{∥}^{2}\right]}^{2}.\;$$

Finally, the partial derivative $\frac{\partial F_{f}\left(e_{1}\right)}{\partial e_{1}}$ is provided by

(53) $$\frac{\partial F_{f}\left(e_{1}\right)}{\partial e_{1}}=\left\{\begin{array}{c} \begin{array}{cc} \frac{1}{\sqrt{\left|A\left(e_{1}\right)\right|}}+\sqrt{\left|A\left(e_{1}\right)\right|}\frac{I-e^{-2\epsilon_{1}}}{e^{-\epsilon_{1}}}sign\left(e_{1}\right) & if\;\left|e_{1}\right|\geq \epsilon_{1} \end{array}\\ \begin{array}{cc} \frac{\pi }{2\epsilon_{1}}A_{1}\cos\left(\frac{\pi e_{1}}{2\epsilon_{1}}\right)+\frac{\epsilon_{1}}{2\pi }A_{2}\cos\left(2\pi \frac{e_{1}}{\epsilon_{1}}\right) & if\;\left|e_{1}\right|\leq \epsilon_{1} \end{array} \end{array}\right..\;$$

#### 3.2.2. Finite-Time Second-Order Sliding Mode Control

Liu et al. [42] presented a finite-time second-order SMC (FTSOSMC) for underwater vehicle trajectory tracking subject to system uncertainties and unknown disturbances. The model expressed in the Body-fixed frame is provided by

(54) $$\dot{\eta }=J\left(\eta \right)v,$$

(55) $$M^{\prime \dot{\upsilon }}=+C^{\prime }\left(\upsilon \right)\upsilon +D^{\prime }\left(\upsilon \right)\upsilon +g^{\prime }\left(\eta \right)=\tau +\tau_{ext},$$

then, the hydrodynamical model can be rewritten as

(56) $$\ddot{\eta }=F\left(\eta ,\dot{\eta }\right)+G\left(\eta \right)\tau +\tau_{d},$$

where

(57) $$F\left(\eta ,\dot{\eta }\right)=M^{-1}\left(-C\dot{\eta }-D\dot{\eta }-g\right),$$

(58) $$G\left(\eta \right)=M^{-1},$$

(59) $$M=M^{\prime }J^{-1}\left(\eta \right),$$

(60) $$C\left(\upsilon ,\dot{\eta }\right)=\left[C^{\prime }-M^{\prime J^{T}\dot{J}}\right]J^{-1}\left(\eta \right),$$

(61) $$D\left(\upsilon ,\dot{\eta }\right)=D^{{\prime J}^{-1}}\left(\eta \right),$$

(62) $$g\left(\eta \right)=g^{\prime }\left(\eta \right).$$

The tracking error is provided by

(63) $$e\left(t\right)=\eta \left(t\right)-\eta_{d}\left(t\right),$$

and the sliding surface is defined as

(64) $$s=\dot{e}+\lambda e,$$

where λ is a positive scalar.

Finally, the control law is provided by

(65) $$\tau =\tau_{e}+M\tau_{s},$$

where

(66) $$\tau_{e}=-G^{-1}\left(F+\lambda \dot{e}-{\ddot{\eta }}_{d}\right),$$

(67) $$\tau_{s}=-k_{1}sign^{b}\left(s\right)-\int_{0}^{t}(k_{2}sign^{2b-1}\left(s\left(\tau \right)\right)+k_{3}s\left(\tau \right)+k_{4}sign^{b}s\left(\tau \right)dt.\;$$

### 3.3. Further Considerations

#### 3.3.1. External Disturbances

Ocean currents can be included as external disturbances in the simulation by using relative velocity, the difference between the actual velocity and the velocity of the ocean current, as described by Fossen [38]

(68) $$\nu_{rel}=\nu -\nu_{oc},$$

where ν is the vehicle velocity and $\nu_{oc}$ is the ocean current velocity.

A generalized vector for an irrotational ocean current velocity is defined as

(69) $$\nu_{oc}={\left[u_{oc},v_{oc},w_{oc},0,0,0\right]}^{T},$$

where $u_{oc}$ is the ocean current velocity from the north, $v_{oc}$ is the ocean current velocity from the east, and $w_{oc}$ is the ocean current velocity from below.

Defining $\alpha_{oc}$ as the angle of attack and $\beta_{oc}$ as the slide slip angle, every element of the velocity vector can be calculated as

$$u_{oc}=\nu_{oc}\cos\alpha_{oc}\cos\beta_{oc},$$

$$v_{oc}=\nu_{oc}\sin\beta_{oc},\;\mathrm{and}$$

(70) $$w_{oc}=\nu_{oc}\sin\alpha_{oc}\cos\beta_{oc}.\;$$

Then, the model provided by Equation (10) can be modified as follows

(71) $$M{\dot{\nu }}_{rel}+C\left(\nu_{rel}\right)\nu_{rel}+D\left(\nu_{rel}\right)\nu_{rel}+g\left(\eta \right)=\tau .\;$$

Zhang et al. [43] performed experiments where the effects of ocean currents on a UUV were measured. The following values of ocean currents velocities are considered for the experimentation in this work

$$u_{oc}=0.75\;\frac{m}{s},$$

$$v_{oc}=0.25\;\frac{m}{s},\;\mathrm{and}$$

(72) $$w_{oc}=0.3\;\frac{m}{s}.\;$$

#### 3.3.2. Saturation Constraints

Since the control signal vector u is limited to −1 ≤ u ≤ 1, its computation was subjected to saturation constraints. For the first four elements of the u vector, corresponding to the four horizontal thrusters $T_{1},\;T_{2},T_{3},$ and $T_{4}$, if the major absolute value is greater than one, $u_{1},u_{2},\;u_{3}$, and $u_{4}$ are computed as

(73) $$u_{i}=\frac{u_{i}}{\max\left(u_{1},u_{2},u_{3},u_{4}\right)},\;$$

with i=1, 2, 3, 4.

Now for the elements $u_{5}$ and $u_{6},\;$corresponding to the vertical thrusters $T_{5}\;$and $T_{6}$, their value is just limited to $-1\;\leq \;u_{j}\;\leq \;1$

(74) $$u_{j}=\left\{\begin{array}{c} u_{j}=1,\;if\;u_{j}>1\\ u_{j}=-1,\;if\;u_{j}<-1 \end{array}\right.\;$$

with j=5, 6. These saturation constraints were applied for the simulations with every controller.

## 4. Results and Discussions

Simulations were performed to test and evaluate the performance of the controllers presented in the previous sections. The effects of ocean currents were not considered at first. Then, the high ocean currents described in Section 3.3.1 were introduced as external disturbances during the mission. The MATLAB source code for the BlueROV2 simulator with the proposed model-free second-order SMC is available as supplementary material Source code S2. The parameters used for the simulations are summarized in Table 6.

### 4.1. Results without Introducing External Disturbances

**Figure 5:**
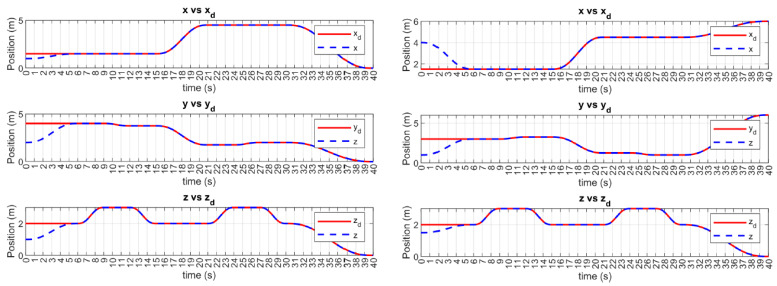
MFSOSMC x, y, and z trajectory tracking (no external disturbances). BlueROV2 #1 (**left**). BlueROV2 #2 (**right**).

Initially, a simulation was performed without considering the effects of ocean currents. The x, y, and z trajectories of BlueROV2 #1 (UUV1) and BlueROV2 #2 (UUV2) are shown in Figure 5. In both cases, convergence to the desired trajectory occurs smoothly at the user-defined time-base $t_{b}$, which was arbitrarily set to 6 s. After reaching the time-base, the vehicles maintained their respective desired trajectories by keeping a practical zero value in the tracking errors. 

**Figure 6:**
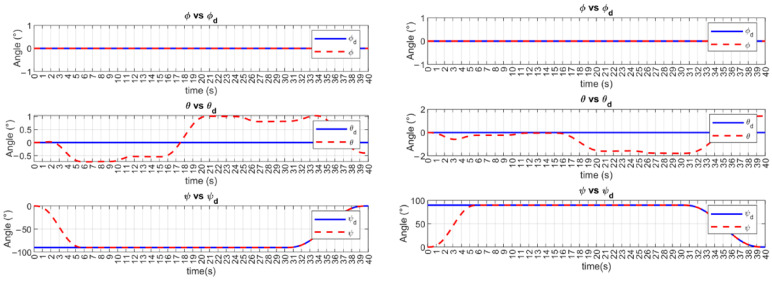
MFSOSMC ϕ, θ, and ψ trajectory tracking (no external disturbances). **Left**: BlueROV2 #1. **Right**: BlueROV2 #2.

The ϕ, θ, and ψ trajectories of UUV1 and UUV2 are shown in Figure 6. A smooth transition to the desired trajectory is observed for ϕ and ψ, in the user-defined time-base. The pitch angle θ shows a slight deviation from the desired value, which is assumed to be caused by the vehicle motion. As stated in Section 2.3, this DoF is not controllable.

**Figure 7:**
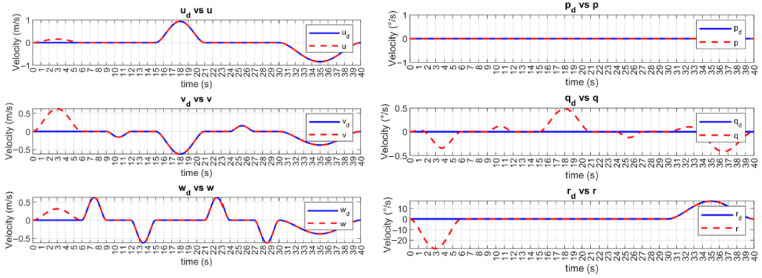
BlueROV2 #1 velocity tracking with the MFSOSMC (no external disturbances).

**Figure 8:**
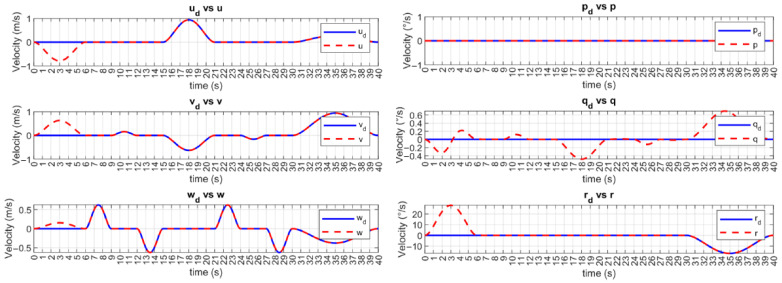
BlueROV2 #2 velocity tracking with the MFSOSMC (no external disturbances).

The desired and controlled velocities are shown in Figure 7 and Figure 8 for UUV1 and UUV2, respectively. The velocities of the vehicles are brought to the desired trajectories at the time-base $t_{b}=6\;s$. Then, the tracking error of these velocities is kept at a practical zero value. Since the angular velocity $\dot{q}$ cannot be controlled, it is assumed that the observed small deviations are due to the vehicle’s motion.

**Figure 9:**
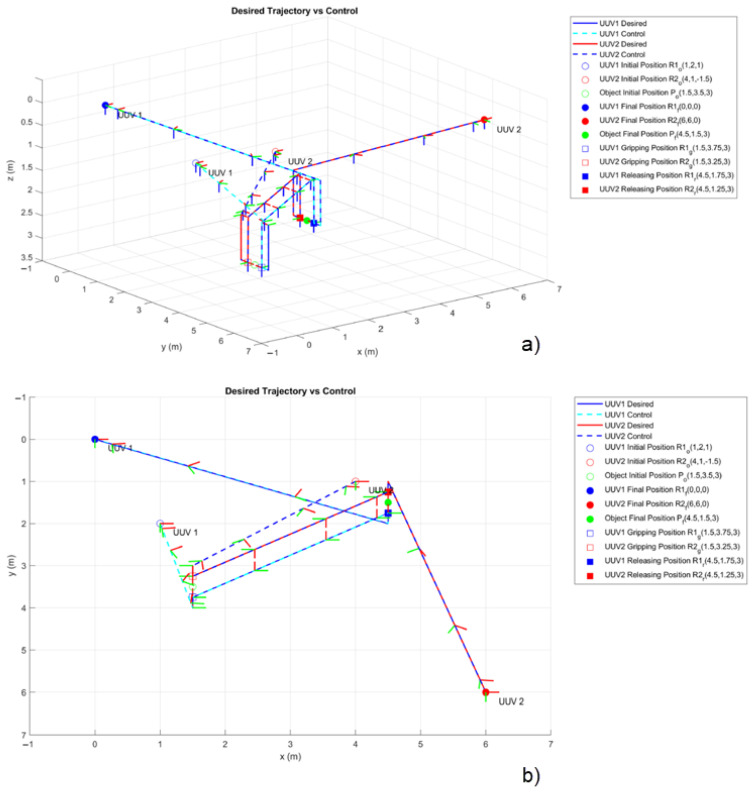
UUV1 and UUV2 trajectory tracking the MFSOSMC in the collaborative manipulation of an object (no external disturbances). (**a**) Isometric view. (**b**) Top view.

The 3D trajectory followed by the vehicles controlled by the MFSOSMC during the designed collaborative manipulation task is shown in Figure 9. The orientation of the vehicles can be observed as their Body-fixed frame is included in the figure. Note that the orientation of the vehicles is initially aligned and later changes to meet the task requirement of facing each other to manipulate the object.

From the results presented earlier, the MFSOSMC guides the vehicles to the desired trajectories in the expected time-base, guaranteeing the vehicles’ synchronization to perform the collaborative manipulation task in a coordinated manner.

**Figure 10:**
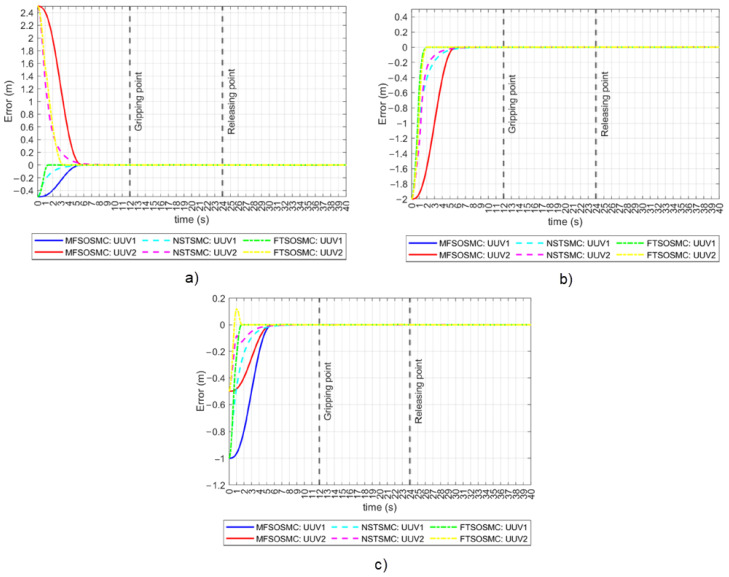
Tracking error for all the controllers (no external disturbances). (**a**) *x*-axis. (**b**) *y*-axis. (**c**) *z*-axis.

The NSTSMC and FTSOSMC presented in Section 3.2 were programmed in the simulator to complete the same collaborative manipulation task. The tracking errors for the x, y, and z positions are shown in Figure 10 for all three controllers. In the tracking error for the x position case, both vehicles converge to a practical zero value simultaneously when driven by the MFSOSMC. Convergence happens at different times for the other controllers as it depends on the initial error condition, which is different for both vehicles. In the case of FTSOSMC, this is more evident as the difference is about two seconds. In the tracking error for the y position case, the proposed MFSOSMC is the only method that achieves coordinated convergence as expected, even when the two vehicles started at the same y coordinate and thus with the same y initial error condition. In the tracking error for the z position case, the two vehicles appear to converge simultaneously. However, the MFSOSMC smoothly controls the vehicles, while the convergence of the other controller is not so smooth and even exhibits an overshoot. This overshoot is not desirable and could harm the vehicle or the object as it could cause the vehicle to collide with the bottom of the water tank or hit the object.

**Figure 11:**
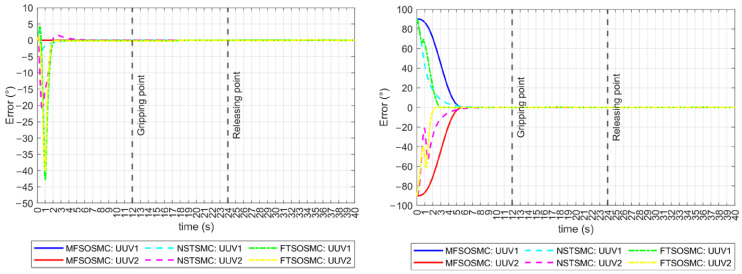
Tracking error for all the controllers (no external disturbances). **Left**: ϕ orientation. **Right**: ψ orientation.

The tracking errors for the ϕ and ψ orientations are shown in Figure 11. In the ϕ orientation case, the initial error condition is zero, which means that the vehicles are already on their reference. The MFSOSMC does not move the vehicles away from the desired ϕ, so the tracking error remains at a zero value. However, NSTSMC and FTSOSMC move the vehicles away from their reference up to 42° and 21°, respectively, which is not desired. In the ψ orientation case, something similar to the x and y tracking errors is observed. The MFSOSMC smoothly tracks the vehicles to the reference with the same time-base, but the NSTSMC and the FTSOSMC converge at different times for UUV1 and UUV2, and the transition is not as smooth because it has ripples. These ripples represent a higher vehicle effort and, therefore, higher energy consumption.

**Figure 12:**
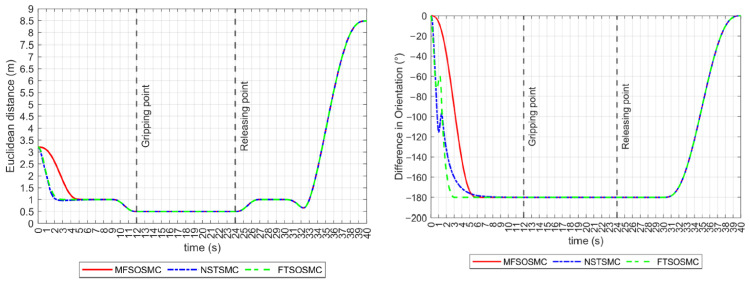
Performance indicators for all the controllers (no external disturbances). **Left**: Euclidean distance in the x axis. **Right**: relative heading orientation.

As described earlier, the distance and relative orientation between vehicles must remain constant at 0.5 m and −180°, respectively, as they approach and manipulate the object. If this is accomplished and tracking errors are kept to zero, the collaborative manipulation task can be successful. These performance indicators are shown in Figure 12. All controllers drive the vehicles to meet these values before the manipulation begins. However, the NSTSMC and FTSOSMC have some difficulty controlling the relative orientation of the vehicles in the transitory.

### 4.2. Simulations with External Disturbances

**Figure 13:**
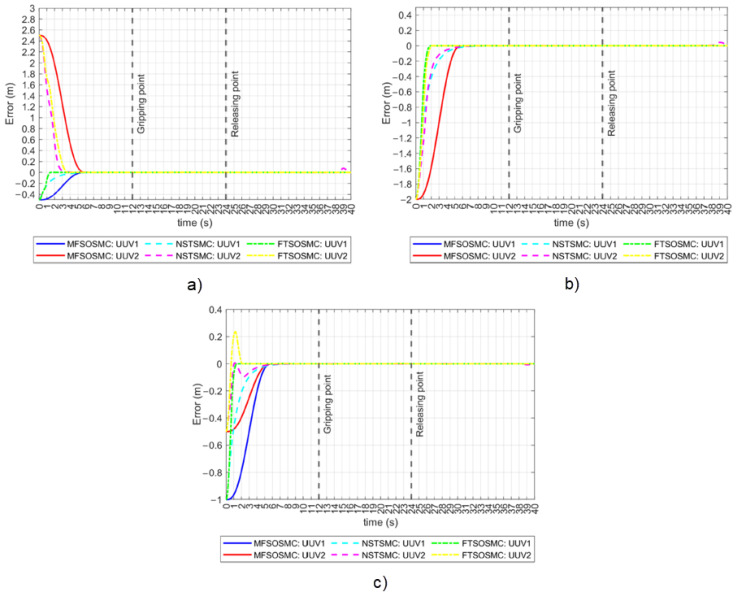
Tracking error for all the controllers (with external disturbances). (**a**) *x*-axis. (**b**) *y*-axis. (**c**) *x*-axis.

Simulations were performed applying the high ocean currents described in the previous sections were considered from the beginning and throughout the collaborative manipulation task. These disturbances remained unknown to the controllers. Tracking errors for the x, y, and z positions are shown in Figure 13. The robustness of the MFSOSMC to strong external disturbances can be observed as it maintains its performance and drives the vehicles to their references smoothly and without any visible problems in the provided time-base. The difficulties of the NSTSMC and the FTSOSMC observed in the previous section were amplified in this case.

**Figure 14:**
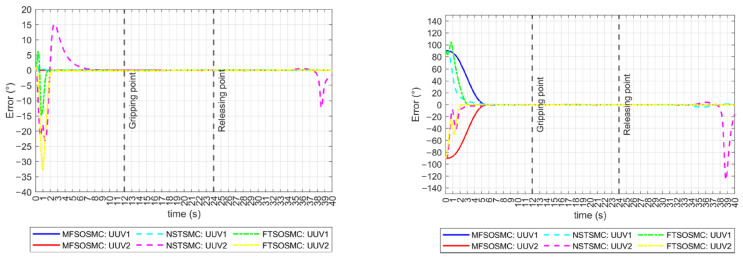
Tracking error for all the controllers (with external disturbances). **Left**: ϕ orientation. **Right**: ψ orientation.

The external disturbances also exacerbated the problems of the NSTSMC and the FTSOSMC in keeping the vehicles on the ϕ=0° reference, as can be seen in Figure 14 (left), because it not only deviates from the reference but also has multiple oscillations. The NSTSMC even lost the reference during the last interval of the task. The MFSOSMC maintains the ϕ=0° reference all the time. The tracking error for the ψ orientation is shown in Figure 14 (right). Multiple ripples are observed for the NSTSMC and the FTSOSMC, while the MFSOSMC smoothly converges to zero. The NSTSMC lost both vehicle references in the last intervals of the task, which is a worse performance than the results without external disturbances.

**Figure 15:**
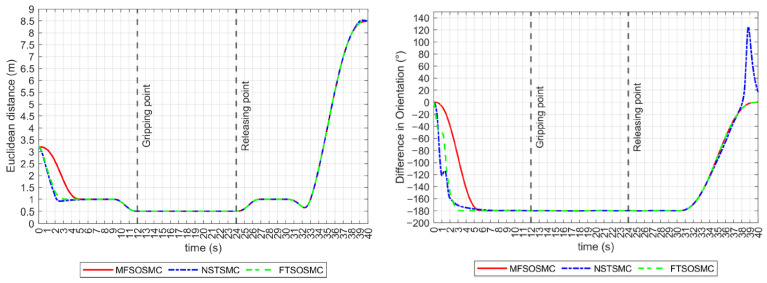
Performance indicators for all the controllers (with external disturbances). **Left**: Euclidean distance in the x axis. **Right**: relative heading orientation.

Regarding the Euclidean distance indicator, all controllers maintained a constant distance of 0.5 m in the *y*-axis during the grasp, transport, and release intervals, as shown in Figure 15 (left). The relative heading orientation is shown in Figure 15 (right), the NSTSMC and the FTSOSMC struggles are noticeable, but the −180° difference is maintained during the manipulation of the object.

**Figure 16:**
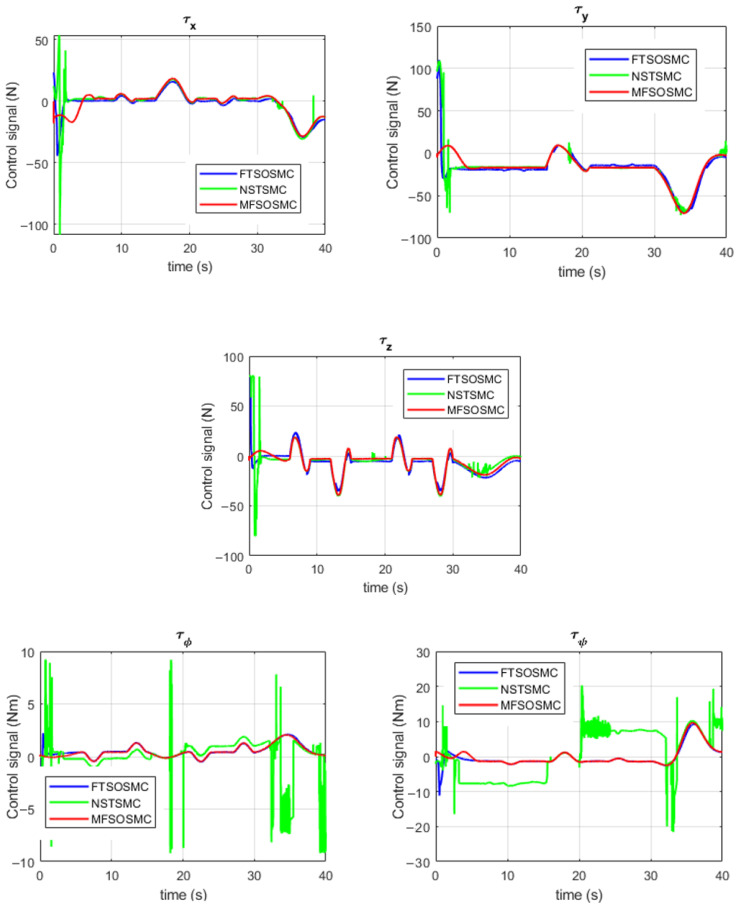
Control signals for UUV1 by all the controllers (with external disturbances).

Another performance indicator is the control signal τ, shown in Figure 16 for the UUV1 control of position and orientation. It can be observed that the control signals of the proposed MFSOSMC start at a small value and increase slightly depending on the manipulation task requirements. The changes in these control signals are smooth, and no chattering is observed at any time. In the case of the FTSOSMC, the initial values of the control signals are considerably higher, which imposes a high demand on the thrusters and higher energy consumption. The NSTSMC provides the worst performance. In this case, the energy demand is higher, and the chattering effect is observed at some intervals.

## 5. Conclusions and Future Work

In this work, a coordinated navigation scheme for two UUVs without communication between them was developed to perform a collaborative manipulation task. A Model-free second-order sliding mode control (MFSOSMC) with finite-time convergence is used to coordinate the navigation of the vehicles so that they converge to their desired trajectories simultaneously. This controller has the advantage that the user can arbitrarily choose the convergence time by simply specifying a time-base parameter $t_{b}.$ This convergence time does not depend on other control parameters, vehicle hydrodynamics, or initial error conditions, as with other state-of-the-art controllers. The coordinated vehicles then perform a collaborative manipulation task of approaching, grasping, lifting, transporting, and releasing an object in a water tank. Numerical simulations validated the performance of the proposed controller, which was compared with two state-of-the-art finite-time controllers, a finite-time second-order sliding mode control (FTSOSMC) and a non-singular terminal sliding mode control (NSTSMC). The results showed superior performance of the MFSOSMC, as the tracking error of all controlled DoFs converged coordinately and smoothly in the predefined time-base and did not deviate from its references, even in the presence of high ocean currents. The FTSOSMC and the NSTSMC had difficulties driving the vehicles to their respective trajectories. Both vehicles converged at different times and exhibited ripples, overshoots, and oscillations. This is highly undesirable because it represents a collision risk that could damage the vehicles or the object and causes a higher energy demand on the thrusters. The proposed MFSOSMC has the best performance in terms of energy consumption observed in the calculated control signals. Higher energy demand was observed in the FTSOSMC and especially in the NSTSMC cases, where chattering also occurred, which is not present in the proposed controller. A constant distance of 0.5 m in the *y*-axis and a relative heading difference of −180° between the vehicles during the manipulation intervals were used as further indicators of the success of the collaborative manipulation task performed by the three controllers, with the MFSOSMC showing the best performance, as described previously. 

In the short term, future work will include a more detailed simulation setup. The coordinated navigation of the vehicles using the proposed MFSOSMC and the collaborative manipulation task can be tested and validated by including more detailed features such as sensors and noise models and considering the object’s physical characteristics. It will require the use of integration testing methods such as MBT. In the long term, this research will address some challenges for validation in an actual experimental setup. Those challenges include:

- Implementing an object identification algorithm;
- Implementing an autonomous path planning algorithm;
- Implementing an autonomous object grasping method;
- Including the physical and geometrical parameters of the object;
- Implementing a method for the localization of the vehicle inside a swimming pool;
- Implementing obstacle avoidance algorithms;
- Implementing an auxiliary control algorithm to deal with vehicle-to-vehicle disturbances.

Validation of coordinated autonomous navigation of multiple (2+) vehicles using the proposed MFSOSMC is also considered for future works.

## Figures and Tables

**Table 1:** Notation for vehicle dynamics according to SNAME.

Name	DoF	Position	Velocity	Force/Moment
Surge	*X* translation	x	u	X
Sway	*Y* translation	y	v	Y
Heave	*Z* translation	z	w	Z
Roll	*X* rotation	ϕ	p	K
Pitch	*Y* rotation	θ	q	M
Yaw	*Z* rotation	ψ	r	N

**Table 2:** Physical parameters of the BlueROV2.

Parameter (Unit)	Value
Dimensions (mm)	457 × 338 × 254
Weight in Air (kg)	11
Net Buoyancy	0.2

**Table 3:** Used parameters for the BlueROV2 simulation.

Parameter (Unit)	Value
$r_{b}\left(m\right)$	${\left[0,0,0\right]}^{T}$
$r_{g}\;\left(m\right)$	${\left[0,0,0.02\right]}^{T}$
$I_{x}\;\left(\mathrm{kg}\cdot m^{2}\right)$	0.16
$I_{y}\;\left(\mathrm{kg}\cdot m^{2}\right)$	0.16
$I_{z}\;\left(\mathrm{kg}\cdot m^{2}\right)$	0.16

**Table 4:** Added mass parameters for the BlueROV2 simulation.

Parameter (Unit)	Value
$X_{\dot{u}}\;\left(\mathrm{kg}\right)$	−5
$Y_{\dot{v}}\;\left(\mathrm{kg}\right)$	−12.7
$Z_{\dot{w\;}}\left(\mathrm{kg}\right)$	−14.57
$K_{\dot{p}}\;\left(\frac{\mathrm{kg}\cdot m^{2}}{\mathrm{rad}}\right)$	−0.12
$M_{\dot{q}}\;\left(\frac{\mathrm{kg}\cdot m^{2}}{\mathrm{rad}}\right)$	−0.12
$N_{\dot{r}}\;\left(\frac{\mathrm{kg}\cdot m^{2}}{\mathrm{rad}}\right)$	−0.12

**Table 5:** Damping parameters for the BlueROV2 simulation.

Parameter (Unit)	Value
$X_{u}\;\left(\mathrm{kg}\right)$	−4.03
$Y_{v}\;\left(\mathrm{kg}\right)$	−6.22
$Z_{w}\;\left(\mathrm{kg}\right)$	−5.18
$K_{p}\;\left(\mathrm{kg}\right)$	−0.07
$M_{q}\;\left(\mathrm{kg}\right)$	−0.07
$N_{r}\;\left(\mathrm{kg}\right)$	−0.07
$X_{u\left|u\right|}\;\left(\frac{N\cdot s^{2}}{m^{2}}\right)$	−18.18
$Y_{v\left|v\right|}\;\left(\frac{N\cdot s^{2}}{m^{2}}\right)$	−21.66
$Z_{w\left|w\right|}\;\left(\frac{N\cdot s^{2}}{m^{2}}\right)$	−36.99
$K_{p\left|p\right|}\;\left(\frac{N\cdot s^{2}}{{\mathrm{rad}}^{2}}\right)$	−1.55
$M_{q\left|q\right|}\;\left(\frac{N\cdot s^{2}}{{\mathrm{rad}}^{2}}\right)$	−1.55
$N_{r\left|r\right|}\;\left(\frac{N\cdot s^{2}}{{\mathrm{rad}}^{2}}\right)$	−1.55

**Table 6:** Parameters used for simulation of the three controllers.

Controller	Parameters
MFSOSMC	$k_{d}=diag\left[800,800,800,800,0,800\right]$,
	$k_{i}=diag\left[1,1,1,1,0,1\right],$
	κ=5,
	$\alpha_{0}=1.01,$
	$\alpha_{c}=25,$
	δ=0.001,
	$t_{b}=6s$.
NSTSMC	β=0.5,
	$\varepsilon_{0}=2,$
	$\gamma =\frac{2}{3},$
	$\varepsilon_{1}=0.01{\left[1,1,1,1,1,1\right]}^{T},$
	$\lambda_{m}=diag\left[0.5,0.5,0.5\right],$
	$\lambda_{p}=diag\left[0.5,0.5,0.5\right],$
	$\alpha_{1}=diag\left[5,5,5,30,30,30\right],$
	$\alpha_{2}=diag\left[5,5,5,30,30,30\right],$
	${\hat{L}}_{m}\left(0\right)={\left[2.5,0.5,\;0.5\right]}^{T},$
	${\hat{K}}_{m}\left(0\right)=diag\left[0.1,0.1,0.1\right].$
FTSOSMC	λ=0.8,
	b=0.7,
	$k_{1}=30,$
	$k_{2}=20,\;$
	$k_{3}=15,$
	$k_{4}=20.$

## Data Availability

Data sharing is not applicable to this article.

## Supplementary Materials

The following supporting information can be downloaded at: https://www.mdpi.com/article/10.3390/s23010239/s1, Video S1: Collaborative manipulation task simulation (MATLAB dynamic Figure), Source code S2: BlueROV2 MATLAB simulator with MFSOSMC.
